# Photoaffinity probe‐based antimalarial target identification of artemisinin in the intraerythrocytic developmental cycle of *Plasmodium falciparum*

**DOI:** 10.1002/imt2.176

**Published:** 2024-02-19

**Authors:** Peng Gao, Jianyou Wang, Chong Qiu, Huimin Zhang, Chen Wang, Ying Zhang, Peng Sun, Honglin Chen, Yin Kwan Wong, Jiayun Chen, Junzhe Zhang, Huan Tang, Qiaoli Shi, Yongping Zhu, Shengnan Shen, Guang Han, Chengchao Xu, Lingyun Dai, Jigang Wang

**Affiliations:** ^1^ State Key Laboratory for Quality Ensurance and Sustainable Use of Dao‐di Herbs, Artemisinin Research Center, Institute of Chinese Materia Medical China Academy of Chinese Medical Sciences Beijing China; ^2^ Department of Pulmonary and Critical Care Medicine, Shenzhen Institute of Respiratory Diseases, and Shenzhen Clinical Research Centre for Geriatrics Shenzhen People's Hospital; First Affiliated Hospital of Southern University of Science and Technology Shenzhen China; ^3^ State Key Laboratory of Antiviral Drugs, School of Pharmacy Henan University Kaifeng China; ^4^ Shandong Academy of Chinese Medicine Jinan China

**Keywords:** antimalarial, artemisinin, chemical proteomics, intraerythrocytic cycle, photoaffinity probe

## Abstract

Malaria continues to pose a serious global health threat, and artemisinin remains the core drug for global malaria control. However, the situation of malaria resistance has become increasingly severe due to the emergence and spread of artemisinin resistance. In recent years, significant progress has been made in understanding the mechanism of action (MoA) of artemisinin. Prior research on the MoA of artemisinin mainly focused on covalently bound targets that are alkylated by artemisinin‐free radicals. However, less attention has been given to the reversible noncovalent binding targets, and there is a paucity of information regarding artemisinin targets at different life cycle stages of the parasite. In this study, we identified the protein targets of artemisinin at different stages of the parasite's intraerythrocytic developmental cycle using a photoaffinity probe. Our findings demonstrate that artemisinin interacts with parasite proteins in vivo through both covalent and noncovalent modes. Extensive mechanistic studies were then conducted by integrating target validation, phenotypic studies, and untargeted metabolomics. The results suggest that protein synthesis, glycolysis, and oxidative homeostasis are critically involved in the antimalarial activities of artemisinin. In summary, this study provides fresh insights into the mechanisms underlying artemisinin's antimalarial effects and its protein targets.

## Highlights

- We identified the targets of artemisinin at different stages of the intraerythrocytic cycle of *Plasmodium falciparum* using a photoaffinity probe of artemisinin.
- Artemisinin can interact with parasite proteins in vivo through both covalent and noncovalent mechanisms.
- Artemisinin may exert its antimalarial effects by interfering with the parasite's protein synthesis, glycolysis, and oxidative homeostasis pathways.
- This study provides fresh insights into the mechanisms underlying artemisinin's antimalarial effects and its protein targets.

## Graphical abstract

Here, both the covalent and noncovalent binding targets of artemisinin at different stages of the intraerythrocytic developmental cycle of *Plasmodium falciparum* were identified using a photoaffinity probe. The results suggest that interfering with the protein synthesis, glycolysis, and oxidative homeostasis pathways of parasites underlies the antimalarial effects of artemisinin. ABPP, activity‐based protein profiling; APP, artemisinin photoaffinity probe; ART, artemisinin; RBC, red blood cell; UV, ultraviolet.

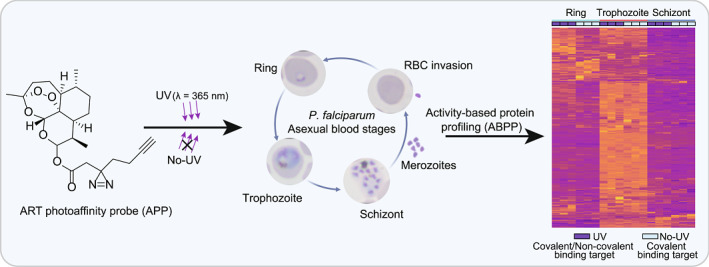

## INTRODUCTION

Malaria is a highly virulent and infectious disease that caused more than 619,000 deaths globally in 2021, largely attributed to *Plasmodium falciparum* (*P. falciparum*) [1]. *P. falciparum* has a complicated and extended life cycle that promotes adaptive growth and impedes malaria control [2]. The 48‐h intraerythrocytic developmental cycle (IDC) consists of several key stages for multiplication, including the ring, trophozoite, and schizont stages [3]. These stages are differentiated by its morphological characteristics [4]. Intraerythrocytic parasites, particularly during the trophozoite stage, are the main target of most antimalarial drugs. Artemisinin (ART) constitutes one of the few antimalarial treatments that exhibits effectiveness throughout the asexual IDC of the parasite [5, 6].

Significant progress have been made in controlling the spread of malaria through the promotion of ART and ART‐based combination therapies (ACTs) [7]. Despite ART still being the recommended the first‐line antimalarial drug, there are escalating concerns about the onset of drug resistance to both ART and ACTs in Southeast Asia and Africa [8, 9]. Concurrently, the antimalarial mechanisms of ART have been extensively investigated using various technologies [10, 11, 12]. Our group has mainly utilized the activity‐based protein profiling (ABPP) approach to profile the targets of ART and revealed that the exceptional antimalarial efficacy of ART results from efficient activation of ART by heme and thereby promiscuous targeting of parasite proteins [13]. While we and other groups have identified covalently bound proteins that are alkylated by free radicals of heme‐activated ART [14, 15], it is likely that certain pharmacological effects of ART may not rely on covalent binding [16, 17, 18, 19]. Indeed, our recent work suggests that ART could interact noncovalently with a handful of parasite proteins in vitro [20]. Previous studies have shown that parasites show different sensitivities to ART at different developmental stages (i.e., the ring, trophozoite, and schizont stages) within the IDC, suggesting that the parasite proteins to which ART binds may vary between stages [21, 22]. Furthermore, the heme‐mediated activation mode of ART also suggests that the ART target proteins may differ between different stages of the IDC, as the rate of hemoglobin (Hb) consumption and heme release by parasites varies at different stages [21, 23]. However, previous studies on ART targets seem to have overlooked these aspects. Therefore, a comprehensive identification of ART targets at different intraerythrocytic stages is necessary to further improve our understanding of the antimalarial mechanisms of ART.

**Figure 1:**
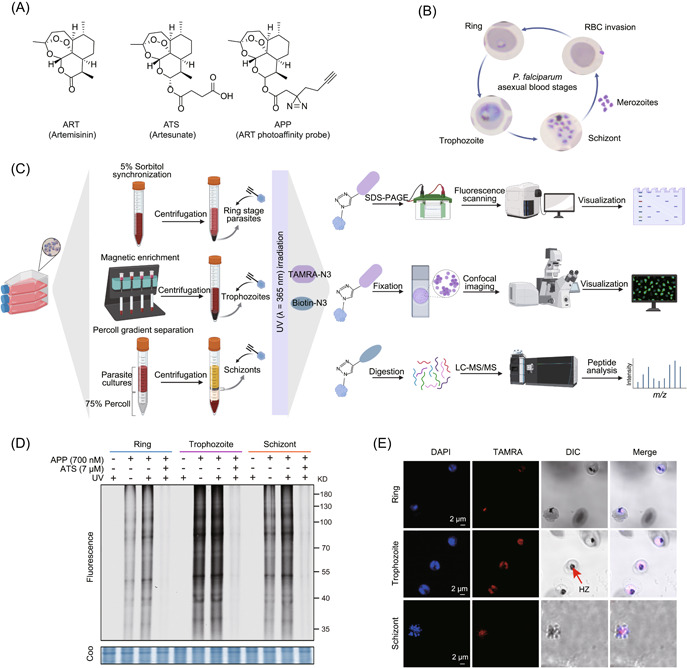
In situ fluorescence labeling of photoaffinity APP in *Plasmodium falciparum* at the ring, trophozoite, and schizont stages. (A) Chemical structures of ART, ATS (an ART derivative with much better aqueous solubility), and APP. (B) Schematic representation of the intraerythrocytic developmental cycle (IDC) of *P. falciparum*. Merozoites invade red blood cells (RBCs) and undergo the ring, trophozoite, and schizont stages to complete the entire IDC. (C) General workflow of the photoaffinity probe APP‐mediated activity‐based protein profiling used to label and identify ART target proteins in vivo. (D) In situ labeling of APP in *P. falciparum* at different stages and competition with excess ATS. (E) Confocal imaging showing the distribution of APP (700 nmol/L) in *P. falciparum* at different stages under ultraviolet irradiation (scale bar = 2 µm). Coo, Coomassie brilliant blue; D, kilo‐Dalton; DAPI, 4′,6‐diamidino‐2‐phenylindole; DIC, differential interference contrast; HZ, hemozoin; LC‐MS/MS, liquid chromatography‐tandem mass spectrometry; nRBC, normal red blood cell; SDS‐PAGE, sodium dodecyl sulfate‐polyacrylamide gel electrophoresis; TAMRA, tetramethyl‐6‐carboxyrhodamine.

In this work, we identified the interacting protein targets (including both covalent and noncovalent binding targets) of ART at the ring, trophozoite, and schizont stages using an ART photoaffinity probe (APP) based on the ABPP technology (Figure 1A). APP has comparable antimalarial efficacy to artesunate (ATS), an ART derivative with much better aqueous solubility. It is also equipped with a diazirine photoactive group and an alkyne reporter moiety that enables covalent crosslinking with the targets upon exposure to 365 nm ultraviolet (UV) irradiation and subsequent click chemistry reaction, which facilitates simultaneous capture of covalent and noncovalent targets. Extensive mechanistic studies were then conducted by integrating target validation, phenotypic studies, and untargeted metabolomics analysis. The results indicate that ART can interact with many parasite proteins in covalent or noncovalent manner, which may collectively contribute to the disruption of parasite's protein synthesis, glycolytic energy supply, and redox homeostasis, ultimately leading to its remarkable antimalarial effects.

## RESULTS

### Fluorescence labeling of APP at different stages of the IDC in *P. falciparum*

To investigate the antimalarial targets of ART in the IDC of *P. falciparum*, we first confirmed the comparable antimalarial efficacy of APP to the original ATS (Supporting Information S2: Figure S1) and then carried out in situ fluorescence labeling of unsynchronized parasites using APP with or without UV irradiation. The results showed that protein labeling by APP was dose‐dependent under both conditions and that UV irradiation had little impact on labeling efficiency (Supporting Information S2: Figure S2). Competition experiments further demonstrated the specificity of the probe. However, given the unique heme‐mediated activation mode of ART and the complexity of the parasite life cycle (Figure 1B), we next examined the interactions between APP and parasite proteins at the ring, trophozoite, and schizont stages of the IDC (Figure 1C).

As shown in Figure 1D, the results indicated that APP interacts with parasite proteins at all three different stages, indicating that ART can be activated by heme throughout the IDC. The fluorescence labeling intensity in the trophozoite stage is significantly stronger than that of the ring and schizont stages (Figure 1D), indicating that ART is more activated during the trophozoite stage and can interact with a greater number of parasite proteins [21]. In live cell imaging experiments, we evaluated the distribution of APP at the ring, trophozoite, and schizont stages using a confocal microscope (Figure 1E). The results showed that the probe labeled a wider range of proteins in trophozoite stage parasites, consistent with the fluorescence labeling results in the gel.

In addition, we found that the fluorescence labeling intensity became stronger after UV irradiation for the parasites at the ring and schizont stages (Figure 1D), indicating that in addition to the irreversible covalent binding mode by free radical reactions, ART can also interact with certain proteins in a noncovalent mode or with the same target protein in both two manners. Nevertheless, there was hardly any significant difference in the fluorescence intensity at the trophozoite stage after UV irradiation (Figure 1D). This may be explained by the fact that parasites consume more Hb during the trophozoite stage, resulting in higher heme concentration, so that ART is rapidly activated and alkylates most, if not all, of the target proteins before the photo‐crosslinking reaction occurs. Another explanation is that the protein conformational change induced by the covalent interaction prevents noncovalent binding.

### Identification of the APP target proteins at different stages of the IDC

**Figure 2:**
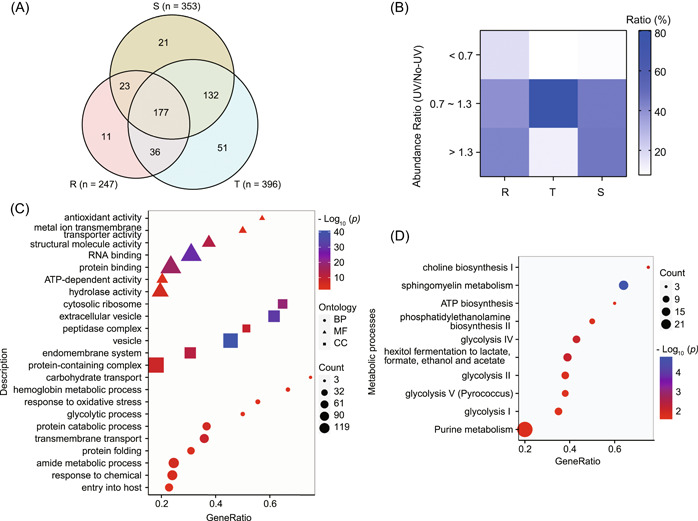
Identification and analysis of artemisinin target proteins using artemisinin photoaffinity probe (APP)‐based activity‐based protein profiling. (A) Venn diagram of the target proteins identified by APP at different stages of *Plasmodium falciparum*. (B) The abundance ratio of target proteins of *P. falciparum* of different stages identified by APP in situ. (C) Metabolic pathway enrichment analysis for all 451 targets. (D) Gene Ontology enrichment analysis for all 451 targets. Three categories including biological process, molecular function, and cellular component were all included. No‐UV, without UV irradiation; R, ring; S, schizont; T, trophozoite; UV, UV irradiation.

Encouraged by the above in vivo fluorescence labeling results, we then attempted to identify the target proteins of APP in *P. falciparum* at the ring, trophozoite, and schizont stages using the pull‐down assay with or without UV irradiation. As shown in Figure 1C, parasites at different stages were incubated with APP or dimethyl sulfoxide (DMSO) control, with or without subsequent UV irradiation. Then, the click chemistry reaction was performed to conjugate labeled proteins with a biotin tag, and the target proteins were enriched with streptavidin beads, digested with trypsin, and identified by high‐resolution mass spectrometry (MS). As shown in Figure 2A and Supporting Information S2: Figure S3A, a total of 451 potential target proteins were identified (Supporting Information S1: Table S1), of which 247, 396, and 353 were identified in the ring, trophozoite, and schizont stages of *P. falciparum*, respectively. Of the 451 targets, 177 were commonly identified in all three stages. The trophozoite stage has the largest number of targets, which may explain why it is the most sensitive stage of the IDC to ART [24]. Analysis of the ratio of abundances of bound proteins clearly shows relatively higher signals for some of the target proteins under UV irradiation than that under non‐UV irradiation during the ring and schizont stages (Figure 2B). However, the effect of UV irradiation on the relative protein abundance was much lower during the trophozoite stage (Figure 2B and Supporting Information S2: Figure S3B). Therefore, the results suggest that in addition to heme activation‐mediated covalent binding to proteins, noncovalent binding of ART to parasite proteins also occur.

### Bioinformatics analysis of the target proteins

Next, we performed Gene Ontology (GO) analysis on these 451 targets, and the results showed that these targets have multiple physiological functions (molecular function analysis) and are involved in diverse biological processes (biological process analysis) at different subcellular localizations (cellular component analysis) within the parasite (Figure 2C). Furthermore, we performed an enrichment analysis of the metabolic processes in which these targets might be involved. As shown in Figure 2D, the results indicated that the targets are mainly involved in several glycolysis‐related metabolic pathways, as well as in the biosynthesis and metabolism of choline, phospholipid, and purine. Protein–protein interaction analysis revealed that the identified targets are mainly involved in multiple processes, such as transcription, protein folding, and catabolism (Supporting Information S2: Figure S4).

**Figure 3:**
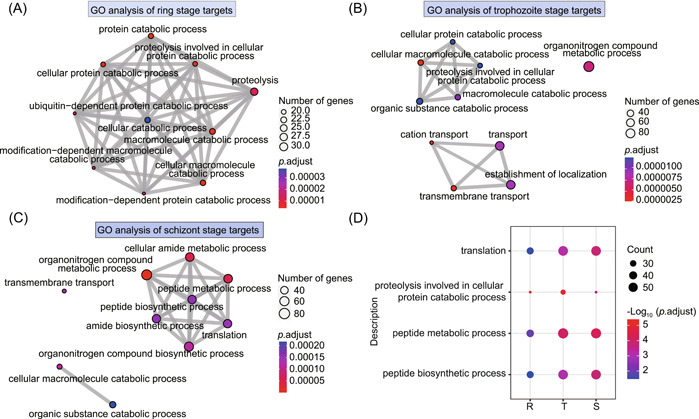
Bioinformatics analysis of the identified artemisinin photoaffinity probe target proteins. (A) Biological process (BP) network analysis of the target proteins at ring stage. (B) BP network analysis of the target proteins at trophozoite stage. (C) BP network analysis of the target proteins at schizont stage. (D) The common biological processes enriched at three different stages of the intraerythrocytic developmental cycle. GO, Gene Ontology.

We also performed GO enrichment analysis on the target proteins of each of the three stages. This analysis revealed that the targets of the ring stage and trophozoite stage are mainly involved in protein catabolic process, proteolysis, and other processes, while the targets of the schizont stage are mainly involved in processes such as peptide metabolism, amide biosynthesis, translation, and others (Figure 3A–C). In addition, we identified four physiological processes, including translation, proteolysis, peptide metabolic, and biosynthetic processes, that were enriched at all three different stages (Figure 3D). However, the extent of targeting varied among the different stages, with the highest level observed in the ring and trophozoite stage parasites. This suggests that the impact of ART on a particular physiological process fluctuates across stages, likely due to varying activation levels of ART at the ring, trophozoite, and schizont stages, and consequently differences in the covalent and noncovalent binding of target proteins.

### ART interferes with the de novo protein synthesis of parasites

**Figure 4:**
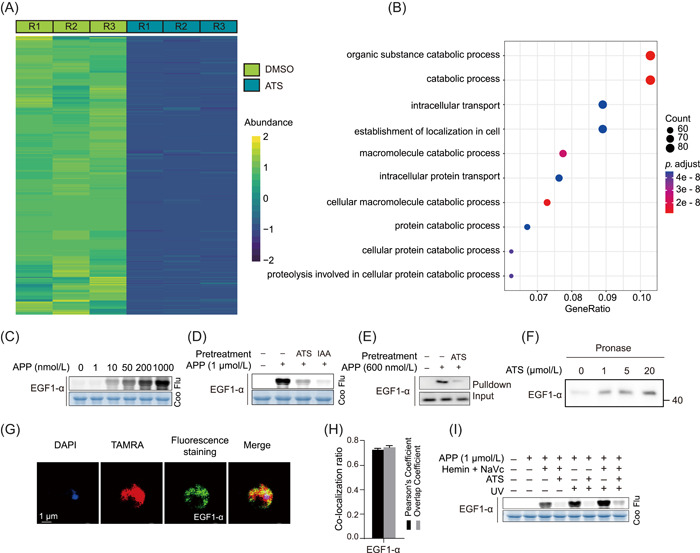
Artemisinin interferes with the protein synthesis in parasites. (A) Heatmap representation of the parasite proteins with reduced synthesis rate after artesunate (ATS) treatment compared to the control group (dimethyl sulfoxide). (B) Gene Ontology‐biological process enrichment analysis of the proteins with reduced synthesis rate. (C) Fluorescence labeling of recombinant *Plasmodium falciparum elongation factor 1‐α* (PfEGF1‐α) (PF3D7_1357000) with artemisinin photoaffinity probe (APP) in a dose‐dependent manner. (D) Preincubation with excess ATS (10 μM) and iodoacetamide (10 μM) competes with APP labeling of recombinant proteins. (E) Pull‐down Western blot analysis validation of APP binding to *PfEGF1‐α* in situ. (F) Validation of ATS to target proteins using the drug affinity responsive target stability method. (G) Representative image of immunofluorescence staining of the co‐localization of APP with *PfEGF1‐α* proteins under ultraviolet irradiation. (H) Quantitative analysis of the co‐localization ratio of (G). (I) Fluorescence labeling of recombinant *PfEGF1‐α* with APP under different conditions. Coo, Coomassie brilliant blue; DAPI, 4′,6‐diamidino‐2‐phenylindole; DMSO, dimethyl sulfoxide; EGF1‐α, elongation factor 1‐α; Flu, fluorescence; NaVc, sodium ascorbate; TAMRA, tetramethyl‐6‐carboxyrhodamine; UV, ultraviolet.

Our target protein analysis indicated that the interference with translation and de novo protein synthesis‐related processes may be essential for the antimalarial effect of ART. During the IDC of *P. falciparum*, parasites digest up to 95% of host cell Hb and release a lot of amino acids for protein synthesis, accompanied by active translation and proliferation [25, 26]. Therefore, we went on to confirm whether protein synthesis in parasite was inhibited by ATS. l‐azidohomoalanine (AHA), a nonradioactive l‐methionine analog that can be incorporated into proteins during protein synthesis, was used to monitor the impact of ATS on the de novo synthesis of new proteins [27]. The azide part of AHA allows a subsequent click chemistry reaction with a biotin‐alkyne probe, which is then enriched by affinity beads, allowing protein identification by high‐resolution MS after digestion. As shown in Figure 4A, the synthesis of 1421 proteins was significantly inhibited after ATS treatment compared to the control. These proteins are mainly involved in organic substance catabolism, intracellular transport, and other critical physiological processes (Figure 4B).

*P. falciparum elongation factor 1‐α* (*PfEGF1‐α*, PF3D7_1357000) was identified as a potential target at all three stages of the IDC (Supporting Information S1: Table S1). We then expressed and purified the recombinant *PfEGF1‐α* protein and verified its interaction with APP. As shown in Figure 4C,D, the labeling of *PfEGF1‐α* by APP under UV irradiation is dose‐dependent and can be competitively bound by excessive amounts of ATS. In addition, similar competitive binding phenomena were also observed in the in situ pull‐down Western blot analysis assay (Figure 4E). Moreover, the drug affinity responsive target stability (DARTS) analysis showed that the *PfEGF1‐α* proteins became more resistant to proteolysis after incubation with ATS (Figure 4F), indicating the specific binding of ATS to *PfEGF1‐α*. The immunofluorescence assay also showed the co‐localization of APP with *PfEGF1‐α* in situ (Figure 4G,H). In addition, we note that after preincubation with the cysteine (Cys) residue blocker iodoacetamide (IAA), the fluorescence labeling intensity of APP decreased to varying degrees (Figure 4D), indicating that Cys may be at least one of the sites where ATS binds to *PfEGF1‐α*. We then examined the binding of *PfEGF1‐α* to ATS under different conditions. As shown in Figure 4I, the fluorescence labeling intensity of *Pf*EGF1‐α under UV irradiation alone is comparable to that under heme and sodium ascorbate alone, indicating that ATS can bind to *PfEGF1‐α* in both covalent and noncovalent mode.

Furthermore, we examined the alterations in the rate of de novo protein synthesis throughout the IDC of *P. falciparum* and the impact of ATS on this process. As shown in Supporting Information S2: Figure S5A,B, the rate of protein synthesis fluctuated, with the highest rate in the trophozoite stage and the lowest rate in the ring stage, and ATS impeded this process throughout the IDC [23]. We also analyzed the changes in proteolysis at the ring, trophozoite, and schizont stages. As indicated by the higher protease activity during the trophozoite and schizont stages compared to the ring stage, the degree of protein hydrolysis is elevated after the ring stage. The results also showed that ATS treatment inhibited protease activities to varying degrees (Supporting Information S2: Figure S5C,D). In addition, many ribosomal proteins were also identified as potential targets of ART (Supporting Information S1: Table S1), further supporting that protein synthesis may be affected. In summary, these results indicate that ATS may bind to related parasite proteins, including *PfEGF1‐α*, throughout the IDC, resulting in the inhibition of protein synthesis and thus antimalarial effects.

### ART interferes with glycolysis in *P. falciparum*

**Figure 5:**
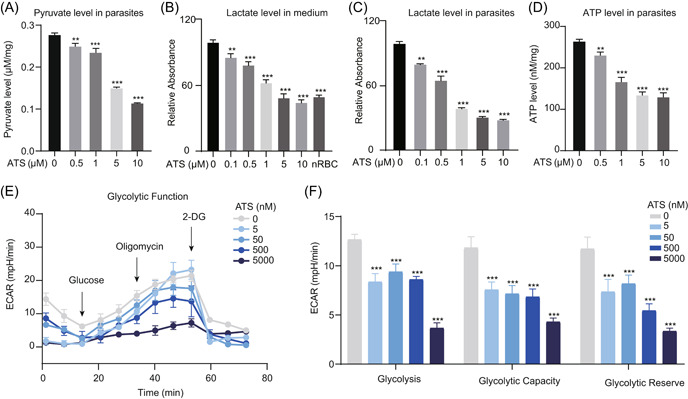
Artesunate (ATS) inhibits the glycolytic process of *Plasmodium falciparum*. (A) ATS reduces pyruvate levels in parasites. (B) ATS reduces the levels of lactate in the culture medium in a dose‐dependent manner. (C) ATS reduces the levels of lactate in the parasites in a dose‐dependent manner. (D) ATS reduces ATP levels in parasites. (E) Inhibition of glycolytic activity by ATS was measured using a Seahorse extracellular flux analyzer. (F) ATS inhibits glycolytic activity in *P. falciparum* in a dose‐dependent manner. All data are averaged from three independent experiments and all values are presented as the mean ± standard error of the mean (SEM).  2‐DG, 2‐deoxy‐glucose; ATP, adenosine 5′‐triphosphate; ECAR, extracellular acidification rate; nRBC, normal red blood cell. **p < 0.01; ***p < 0.01.

As previously analyzed, metabolic pathways relating to glycolysis and energy metabolism were predominantly enriched in the identified targets, which was expected given that *P. falciparum* absorbs a significant amount of glucose during the IDC and relies heavily on glycolysis to provide energy for rapid growth and proliferation [28]. This also leads to an increase in the production of pyruvate and lactate [29, 30]. The glycolysis pathway has been identified as an essential pathway for ART to produce antimalarial activity [14, 31]. Therefore, we went on to examine the impact of ART on parasite glycolysis. First, we measured the level of pyruvate in parasites, as well as the level of lactate in both parasites and culture medium. As the drug concentration increased, the levels of pyruvate and lactate decreased significantly (Figure 5A–C). ATP levels showed the same decreasing trend (Figure 5D). We then further evaluated the impact of ATS on the glycolytic activity of intraerythrocytic parasites by measuring the extracellular acidification rate (ECAR) [32, 33]. As shown in Figure 5E,F, the glycolysis process and glycolysis capacity of the parasites were significantly attenuated by ATS in a dose‐dependent manner, consistent with the reduced lactate level.

Considering that glycolysis is one of the most fundamental metabolic pathways, some highly active and expressed enzymes related to it are considered as promising targets for antimalarial drugs [34]. In our previous study, *P. falciparum l‐lactate dehydrogenase* (*PfLDH*, PF3D7_1324900), *triosephosphate isomerase* (*PfTIM*, PF3D7_1439900), and *glyceraldehyde‐3‐phosphate dehydrogenase* (*PfGAPDH*, PF3D7_1462800) were identified as potential targets for ART [20], and they were also identified in this work. We first validated their interaction with ATS using DARTS (Supporting Information S2: Figure S6A–C), and then measured the inhibitory effect of ATS on their enzymatic activities, and the results showed that the half‐maximal inhibitory concentrations were at low micromolar concentrations (Supporting Information S2: Figure S6D–F). In addition, we also tried to identify the possible covalent binding sites of ATS to recombinant *PfGAPDH* using high‐resolution MS. The results indicated that the glutamic acid residue 59 might be a binding site, which was supported by molecular docking simulation (Supporting Information S2: Figure S6G). The above results suggest that ATS may bind to glycolytic enzymes, including *PfLDH*, *PfGAPDH*, and *PfGAPDH*, to inhibit their activities, thereby interfering with the glycolytic process of parasites, and thus exerting antimalarial effects during the IDC.

### ART interferes with the redox homeostasis of parasites

**Figure 6:**
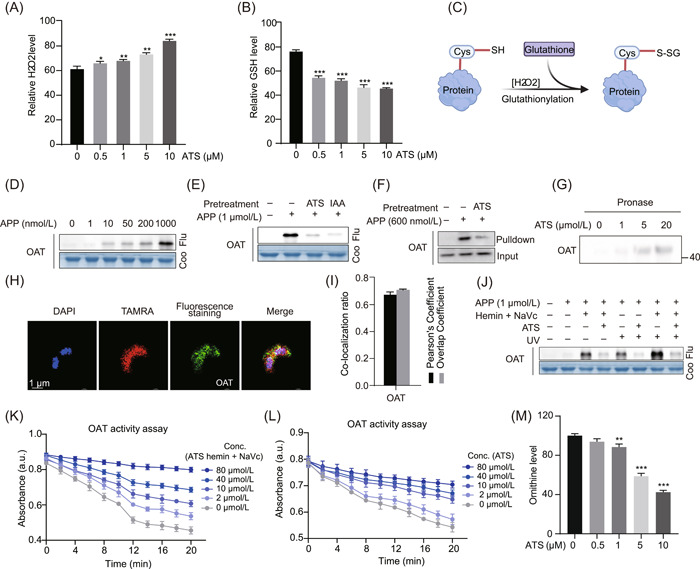
Determination of hydrogen peroxide (H_2_O_2_), glutathione (GSH), and validation of the targeting of artesunate (ATS) to *Plasmodium falciparum ornithine aminotransferase* (*PfOAT* ) (PF3D7_0608800). (A) ATS increases the level of H_2_O_2_ of *P. falciparum*. (B) ATS decreases the level of GSH of *P. falciparum*. (C) Schematic diagram of protein S‐glutathionylation. (D) Fluorescence labeling of recombinant *PfOAT* (PF3D7_1357000) with APP in a dose‐dependent manner. (E) Preincubation with excess ATS (10 μM) and iodoacetamide (IAA) (10 μM) competes with APP labeling of recombinant proteins. (F) Pull‐down Western blot analysis validation of APP binding to *PfOAT* in situ. (G) Validation of ATS to target proteins using the drug affinity responsive target stability (DARTS) method. (H) Representative image of immunofluorescence staining of the co‐localization of APP with *PfOAT* proteins under ultraviolet irradiation. (I) Quantitative analysis of the co‐localization ratio of (G). (J) Fluorescence labeling of recombinant *PfOAT* with APP under different conditions. (K) ATS inhibits the enzymatic activities of recombinant *PfOAT* in vitro in a dose‐dependent manner with heme activation. (L) ATS inhibits the enzymatic activities of recombinant PfOAT in vitro in a dose‐dependent manner without heme activation. (M) ATS reduces ornithine levels in a dose‐dependent manner. All data are based on at least three independent biological replicates and are presented as the mean ± SEM. a.u., arbitrary unit; conc., concentration; Coo, Coomassie brilliant blue; Flu, fluorescence. *p < 0.05; **p < 0.01; ***p < 0.01.

During the IDC, parasites consume significant amounts of glucose for energy supply and degrade lots of Hb for growth and proliferation. This metabolic process generates numerous oxidative byproducts, including superoxide anion free radicals (O_2_˙^−^) and hydrogen peroxide (H_2_O_2_). This resulting oxidative damage places the parasites in a state of high oxidative stress [35, 36]. Antioxidant activity and oxidative stress pathways were notely enriched in the GO analysis of all targets, as shown in Figure 2D. The parasite's antioxidant defense system mainly comprises glutathione (GSH) and various thioredoxin‐dependent proteins [37]. GSH is the most abundant low‐molecular‐weight redox‐active thiol in parasites, which can neutralize oxidative by‐products such as H_2_O_2_, effectively maintaining the intracellular reducing environment [36, 38]. In addition, fluctuations in GSH levels may also affect the drug sensitivity of ART [39]. Here, we simultaneously detected the levels of H_2_O_2_ and GSH in parasites. As shown in Figure 6A,B, H_2_O_2_ increased significantly after the treatment with increasing ATS, while GSH showed the opposite trend. These results suggest that ATS may cause oxidative damage to parasites by disrupting their redox homeostasis, which is also supported by the changes in malondialdehyde and peroxidase activity in parasites after ATS treatment (Supporting Information S2: Figure S7A,B). In addition, we showed that activated ATS can undergo covalent binding with the thiol group of GSH in vitro (Supporting Information S2: Figure S8). Therefore, ATS may augment oxidative damage in parasites by reducing the levels of redox‐active substances such as GSH.

The protein S‐glutathionylation reaction can affect the function of modified proteins (Figure 6C). A notable example is *P. falciparum ornithine aminotransferase* (*PfOAT*, PF3D7_0608800), which directly affects polyamine synthesis by regulating ornithine levels and is involved in the regulation of glucose metabolism and cell proliferation [40, 41]. The active site Cys residues 154 and 163 of *PfOAT* can be modified by S‐glutathionylation to form disulfide bond, resulting in impaired substrate binding and hence catalytic activity [42]. Here, *PfOAT* was identified as a potential target in all three stages of the IDC and has been previously identified in other studies [13, 43]. We first validated the specific binding of APP to *PfOAT* in vitro (Figure 6D–I) and the existence of both binding modes (Figure 6J). Enzyme activity experiments showed that ATS can significantly inhibit its enzyme activity in both heme‐activated and nonactivated states (Figure 6K,L), as well as decrease ornithine levels in parasites (Figure 6M). These results all clearly indicate that covalent and noncovalent binding modes may be jointly involved in the interaction of ATS with *PfOAT*.

Furthermore, we also validated the interaction of APP with two important peroxidative proteins involved in the maintenance of redox homeostasis in parasites—*P. falciparum thioredoxin peroxidase 1* (*PfTrx‐Px1*, PF3D7_1438900) and *1‐Cys peroxiredoxin* (*Pf1‐CysPXn*, PF3D7_0802200) (Supporting Information S2: Figure S7C) [44, 45], which were also among the identified potential binding targets in this work (Supporting Information S1: Table S1). The results suggest that ATS may also bind to these proteins both covalently and noncovalently, potentially affecting the redox homeostasis of parasites.

### ART interferes with the metabolic process of *P. falciparum*

**Figure 7:**
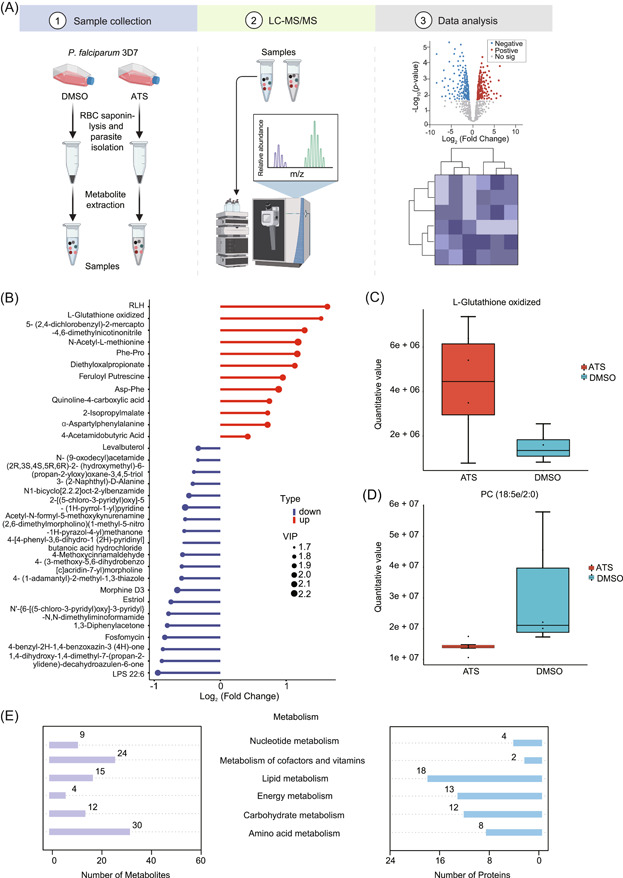
Untargeted metabolomics analysis of *Plasmodium falciparum* treated with artesunate (ATS) versus control. (A) Schematic workflow for the untargeted metabolomics study showing sample collection, liquid chromatography‐tandem mass spectrometry identification, and data analysis. (B) Stem plot showing the metabolites with significant differences. (C) The levels of oxidized l‐glutathione after ATS treatment compared with the control group (dimethyl sulfoxide [DMSO]). (D) The levels of oxidized PC (18:5e/2:0) after ATS treatment compared with the control group (DMSO). (E) Metabolites and related proteins involved in enriched metabolic pathways. Data are presented as the mean ± SEM. LC‐MS/MS, liquid chromatography‐tandem mass spectrometry; PC, phosphatidylcholine; RBC, red blood cell; VIP, variable importance in projection.

When parasites are under significant stress and cannot compensate, cascading reactions that affect multiple systems including metabolic pathways occur [46]. Given the considerable inhibitory effect of ATS on the glycolytic pathway of parasites and the interference of ATS with various physiological processes of parasites, we next investigated the impact of ATS on the metabolome of parasites using untargeted metabolomics analysis (Figure 7A).

First, we performed partial least‐squares discriminant analysis to evaluate the metabolic differences between the control group and the ATS‐treated parasites (Supporting Information S2: Figure S9A) [31]. The results showed a notable distinction between the two groups, indicating that ATS caused significant changes in the metabolism of parasites. A total of 818 metabolite characteristics were identified from the untargeted metabolomics map and were used for further analysis. Variable importance in projection (VIP) > 1, *p* < 0.05, and fold change (FC) > 1.2 were used to screen for significantly altered metabolites, resulting in 42 differentially expressed metabolites (Supporting Information S2: Figure S9B and Supporting Information S1: Table S2). Among them, 30 metabolites were downregulated, whereas 12 were upregulated (Figure 7B and Supporting Information S2: Figure S10). The observed increase in the level of oxidized glutathione was significant (Figure 7C) and consistent with the measurement results in Figure 6B. In addition, a significant reduction in lipid metabolism‐associated products including LPS and phosphatidylcholine was observed (Figure 7D), consistent with the scenario that parasites require large amounts of lipid substances at the intracellular stage to construct new cell membranes to support the growth of newborn parasites [47, 48, 49]. Finally, we mapped the APP target proteins onto the enriched metabolic processes and identified some of the potentially most important target proteins involved in each metabolic process (Figure 7E and Supporting Information S1: Table S3). Due to the haploid nature of *Plasmodium* and the fact that many parasite proteins are encoded by single‐copy genes [50, 51], it is reasonable to assume that most of the mapped proteins are the key targets related to ATS's impact on parasite metabolism.

In summary, our untargeted metabolomics analyses suggest that ATS can extensively interfere with multiple metabolic pathways of intraerythrocytic parasites, which may directly or indirectly converge on the collective antimalarial effects of ART.

## DISCUSSION

ART is the sole frontline antimalarial drug that has yet to develop widespread resistance, and one of the few drugs that is effective against malaria throughout the entire asexual IDC of the parasite. Elucidation of the exact antimalarial mechanisms of ART is urgently needed to optimize current ART drug regimens and address ART resistance, even if there are temporarily mitigatory plans [52]. Research on the antimalarial mechanisms of ART has been progressing slowly, due to the complex life cycle of malaria parasites and the intricate mechanism of action (MoA) of the drug. Currently, most studies focus on the alkylation and covalent interaction impact of activated ART on parasite proteins and the effects of noncovalent binding of ART with parasite proteins in the antimalarial process, and its biological significance remains ambiguous, we feel that other modes of interaction should not be overlooked, therefore in‐depth studies are necessary.

In this work, we comprehensively identified the protein targets of ART at the ring, trophozoite, and schizont stages of the IDC of *P. falciparum* in situ using an active photoaffinity probe. We investigated the binding mode and conducted a series of validation experiments. ART primarily forms covalent bonds with proteins upon activation by heme, but noncovalent binding events also exist. On the one hand, there are differences in the type and importance of pathways that contribute to antimalarial effects at different life stages of *P. falciparum*, which may be due to differences in target protein synthesis that align with the stage of parasite growth. On the other hand, there are differences in the modes of binding (covalent and noncovalent binding) to the target protein, which may be due to different levels of heme in different parasite stages, thereby altering the levels of ART activation. Our work provides important experimental evidence for a comprehensive investigation of the covalently and noncovalently bound targets that are potentially related to the antimalarial mechanism of ART. We note, however, that given the rapid activation of ART in parasites, it is unclear how much of the nonactivated form of ATS is reversibly bound and the extent of its inhibition on the target protein function, which still requires further in‐depth study.

We then performed bioinformatic analysis of all the targets and validated several critical proteins/pathways that may be involved in the antimalarial effect. Our results suggest that ART may exert its antimalarial effect by blocking parasite protein synthesis, interfering with the glycolytic energy supply pathway, and disrupting redox‐related processes. We have also performed untargeted metabolomics to investigate the effect of ART on the overall metabolic process in parasites. However, more research is needed to determine the most critical antimalarial targets and pathways and the relationships between them. Finally, since the MoAs of many antimalarial drugs remain largely elusive, our work suggests that combining phenotyping, target identification, and metabolomics is a promising strategy to elucidate these MoAs.

## CONCLUSION

Overall, this work presents a wealth of data as a valuable resource for the target identification of ART and provides new insights for the study of the antimalarial mechanism of ART, which is necessary to optimize current antimalarial drug regimens and offer novel solutions to mitigate ART resistance.

## METHODS

### Parasite culture

Continuous culture of the *P. falciparum* 3D7 strain was based on the established method with slight modifications [53]. Briefly, parasites were cultured in complete medium supplemented with 2% healthy human erythrocytes and maintained under standard conditions with slight shaking [43]. Parasitemia was evaluated daily using Giemsa‐stained thin blood smears. To prepare highly synchronized parasites at the ring, trophozoite, and schizont stages, parasites were tightly synchronized twice by treatment with 5% sorbitol to obtain ring‐stage parasites, enriched by magnetic separation (MACS CS columns; Miltenyi Biotech) to obtain trophozoite‐stage parasites, and schizont‐stage parasites were isolated by centrifugation over 70% (V/V) Percoll cushions (Novon Scientific) [54].

### Fluorescence labeling in vivo of *P. falciparum*

Fluorescence labeling in vivo was carried out according to our previously described protocol, with minor modifications [43]. Briefly, parasites were cultured in six‐well plates at 5% parasitemia. After incubation with 700 nM APP or an equivalent volume of vehicle (DMSO, final concentration less than 0.1%) for 4 h, the plates were exposed or not to 365 nm UV irradiation for 10 min on ice. For competition, the parasites were pretreated with excess ATS for 1 h, followed by APP for 4 h. After parasite release, the soluble parasite protein was extracted, and equal amounts of protein lysates (20 µg) were used for fluorescence labeling with tetramethyl‐6‐carboxyrhodamine azide (TAMRA‐azide) fluorescent tag (50 μM) through the click chemistry reaction (CuAAC). After the reaction, the proteins were separated by sodium dodecyl sulfate‐polyacrylamide gel electrophoresis (SDS‐PAGE) electrophoresis.

### Target identification through pull‐down assay

The pull‐down assay was performed as previously reported [13]. Briefly, highly synchronized parasites isolated at different life stages were treated with 700 nM APP or an equal volume of DMSO for 4 h. The samples were then irradiated with 365 nm UV for 10 min on ice or not. After irradiation, the soluble parasite proteins were extracted and quantified as described above. Immediately thereafter, CuAAC was performed to conjugate the biotin tag to the proteins with biotin azide. After precipitation and resolubilization, proteins were enriched with NeutrAvidin beads (Thermo Fisher). For target identification, the enriched proteins were reduced and alkylated with dithiothreitol (DTT) and IAA, respectively, and digested with trypsin (Promega) at 37°C overnight. The supernatants were collected, desalted, and dried for subsequent liquid chromatography‐tandem mass spectrometry (LC‐MS/MS) analysis.

For western blot analysis of target validation, the enriched targets were subsequently separated by SDS‐PAGE. Proteins were then transferred to polyvinylidene fluoride membranes, incubated with streptavidin–horseradish peroxidase or appropriate primary and secondary antibodies, and visualized using electrochemiluminescence.

### LC‐MS/MS measurement and protein identification

LC‐MS/MS analysis for target identification was performed as previously described [20]. Data acquisition was carried out with Xcalibur (version 4.2.4) in a data‐dependent acquisition mode. All spectra were detected with MS spectra between 300 and 1500m/z at a resolution of 60,000. The acquired spectra were analyzed using Sequest HT against the *P. falciparum* 3D7 protein database (PlasmoDB‐60, https://plasmodb.org/plasmo/app/downloads/release-60/P.falciparum3D7/fasta/data/). Static and dynamic modifications were set as previously described [20].

### Intracellular imaging assay of APP

The imaging assays were performed as described in a previous study [43]. Parasites were treated with APP or DMSO for 30 min, fixed, and dropped onto poly‐d‐lysine‐coated coverslips, and permeabilized. Click reaction with TAMRA‐azide was then performed. Subsequently, the coverslips were transferred to a 4′,6‐diamidino‐2‐phenylindole‐coated slide and imaged using a TCS SP8 SR confocal microscope.

### Effect of ATS on de novo protein synthesis of *P. falciparum*

The AHA labeling was performed to identify ATS‐inhibited de novo proteins. Briefly, unsynchronized parasites were treated with ATS (700 nM) or vehicle control for 6 h culture in l‐methionine‐free medium containing AHA after 30 min depletion of intracellular methionine. Subsequently, parasite‐soluble proteins were extracted, and CuAAC was performed to conjugate the biotin tag to proteins with biotin‐alkyne and enriched by pull‐down assay.

To examine the effect of ATS on the inhibition of de novo protein synthesis, the experiments were performed as previously described and with minor modifications [27, 55]. Briefly, parasites were highly synchronized twice by treatment with 5% sorbitol to ring stage. Infected erythrocytes were washed three times with medium without serum and l‐methionine. Infected erythrocytes were then cultured with l‐methionine‐free culture medium for 30 min to deplete the intracellular methionine reserves. After the depletion, the medium was replaced with l‐methionine‐free medium containing AHA, and incubated with ATS (100 nM) or equal volumes of vehicle control for 5 h. Parasite samples were harvested at every 5 h intervals. Soluble proteins were then extracted for click reaction and separated by SDS‐PAGE for fluorescence scanning.

### Drug affinity responsive target stability assay

The drug affinity responsive target stability (DARTS) method was used to detect the binding of ATS to target proteins [56]. Briefly, recombinant proteins (5 μM) were treated with a series of ATS and incubated at room temperature for 2 h. Then, 1 μg/mL of pronase was added and incubated for 10 min at room temperature. After the addition of loading buffer, the reaction was terminated at 95°C, followed by SDS‐PAGE electrophoresis for immunoblotting.

### Determination of the proteolysis activity

The protease activity of parasites at the ring, trophozoite, and schizont stages was measured using the fluoro protease assay kit (G‐Biosciences; C006028). Briefly, highly synchronized parasites at different life stages were incubated with ATS (100 nM) for 4 h. Parasites were then collected, released from infected blood cells, and lysed by sonication on ice to extract soluble parasite proteins. After bicinchoninic acid quantification, aliquots of proteins were taken and incubated with protease substrate at 37°C. After the incubation, precipitating agent was added and incubated for another 10 min, followed by centrifugation at 12,000 relative centrifugal force (*g*) for 5 min. The supernatants were then transferred to new tubes and mixed with detection buffer. The optical density absorbance was measured at 570 nm.

### Determination of lactate levels

Parasites were treated with different concentrations of ATS for 4 h. After incubation, the supernatant medium and infected red blood cells were collected. Lactate levels were then determined according to the instructions of the Lactate Content Assay Kit (Solarbio; BC2235).

### Determination of pyruvate levels

The pyruvate levels of parasites were determined using the Pyruvate Content Assay Kit (Solarbio; BC2205). In brief, parasites were treated with a serial concentration of ATS for 4 h, then lysed by sonication with the pyruvate extraction buffer and rested for 30 min. After centrifugation at 800*g* for 10 min at room temperature, the supernatants were collected for further detection of pyruvate levels according to the manufacturer's instructions. The OD absorbance was detected at a wavelength of 520 nm.

### Extracellular flux analysis

The ECAR of *P. falciparum* was measured using the Glycolysis Stress Test Kit on an Agilent Seahorse XFe96 Analyzer. All assays were conducted as previously described and according to the manufacturer's instructions with minor modifications [32, 33]. Briefly, the sensor cartridge was hydrated overnight at 37°C. *P. falciparum* parasites were incubated with increasing concentrations (0–20 µM) of ATS and released with 0.01% saponin. The parasites were then resuspended in Seahorse XF RPMI‐1640 medium (Agilent Technologies) containing 1 mM glutamine (Agilent Technologies), and seeded into a Seahorse miniplate precoated with 0.01% (w/w) polylysine. Subsequently, other assay media (10 mM glucose, 1 µM oligomycin, and 50 mM 2‐deoxy‐2‐[(7‐nitro‐2,1,3‐benzoxadiazol‐4‐yl) amino]‐d‐glucose) were added sequentially to the corresponding wells, and the plate was loaded into Seahorse XFe96 Analyzer for measurement.

### Identification of ATS binding site

As we described previously [20], 10 μM recombinant protein was incubated with 100 μM ATS, 20 μM hemin, and 200 μM sodium ascorbate  for 4 h. Then, the samples were reduced and alkylated with DTT and IAA and digested at 37°C overnight. The peptides were then desalted and dried in a centrifugal vacuum evaporator before LC‐MS/MS analysis as described above.

### Molecular docking model

Docking simulation was performed using Molecular Operating Environment (version 2019 0102) with parameters previously described. The chemical structure of ATS was downloaded from PubChem (PubChem ID: 6917864). The protein structure of *PfGAPDH* (PDB:1T24) was downloaded from the PDB database and prepared using the QuickPrep module.

### Detection of H_2_O_2_ and glutathione

The hydrogen peroxide assay kit (Beyotime) and the total glutathione assay kit (Beyotime) were used to detect H_2_O_2_ and GSH levels in parasites. In brief, parasites were inoculated into 6‐well plates and incubated with different concentrations of ATS (0, 0.5, 1, 5, and 10 μM) in an incubator at 37°C for 4 h. The parasites were released with 0.05% saponin, and the levels of H_2_O_2_ and GSH were detected according to the kit instructions.

### OAT activity assay

The OAT activity assay was performed as we described previously [43]. Purified *PfOAT* protein (5 μg) was incubated with different concentrations (0–80 μM) of ART for 1 h, before being transferred to a 96‐well plate. The substrate nicotinamide adnenine dinucleotide and reaction buffer were then added according to the manufacturer's instructions of the assay kit (Solarbio; BC4405), followed by continuous detection of absorbance at 340 nm.

### Expression and purification of recombinant proteins

The coding sequences of the target proteins were obtained from the PlasmoDB database (http://PlasmoDB.org). The sequences were synthesized and cloned into the pET‐28a (+) vector by Sangon Biotech. The recombinant proteins were expressed in *Escherichia coli* BL21 (DE3) and induced by isopropyl‐β‐d‐thiogalactoside. Then, the cell lysates containing recombinant proteins were extracted under a pressure of 1200 bar, loaded onto Ni‐NTA chromatography, and eluted to obtain recombinant proteins.

### Fluorescence labeling of recombinant proteins in vitro

The recombinant target proteins were validated as previously described [20]. For the dose‐dependent assay, equal amounts of recombinant proteins (2 μg) were incubated with increasing concentrations of APP for 4 h. For the competition assays, recombinant proteins were pretreated with excess ATS or IAA for 2 h before incubation with APP for another 2 h. Then, CuAAC was performed with TAMRA‐azide. The other steps were the same as those described above.

### Sample preparation for metabolite profiling

The *P. falciparum* 3D7 strain (unsynchronized) was cultured as described above, diluted to 10% parasitemia at 2% hematocrit, and then incubated with ATS (700 nM) and equal volumes of vehicle control (0.1% DMSO) for 6 h under standard conditions. Parasites were then collected and released from the erythrocytes. Then, parasites were immediately dropped into liquid nitrogen and stored at −80°C for further metabolite profiling within 2 days.

### Untargeted metabolomics analysis using UHPLC‐MS/MS and data processing

Metabolite extraction of parasites (2 × 10^8^ cells/per sample) was performed as previously described [57]. Samples were injected into a Hypersil Gold column. The eluents were 0.1% formic acid (A) and methanol (B) (positive polarity mode) and 5 mM ammonium acetate (pH 9.0) (A) and methanol (B) (negative polarity mode). All experiments were repeated independently at least three times. Raw data generated by ultra‐high performance liquid‐chromatography tandem MS were processed using Compound Discoverer 3.1 (Thermo Fisher). Peak intensities were normalized to the total spectral intensity and matched against the mzCloud (https://www.mzcloud.org/), mzVault, and MassList databases. *p* value < 0.05, FC > 1.2 or FC < 0.833, and VIP > 1 were set as the significance thresholds to screen for differential metabolites.

### Statistical analysis

GraphPad Prism 8.3 was used for statistical analysis. All data are based on at least three independent biological replicates and are presented as the mean ± standard error of the mean. Statistical significances were analyzed by one‐way analysis of variance, and a *p* value of less than 0.05 was considered significant difference.

## AUTHOR CONTRIBUTIONS

Jigang Wang, Lingyun Dai, Chengchao Xu, and Guang Han conceived the project and designed the experiments. Peng Gao, Jianyou Wang, and Honglin Chen performed the major experiments. Peng Gao, Chen Wang, and Jiayun Chen performed bioinformatic analysis and data visualization. Huimin Zhang, Yongping Zhu, and Shengnan Shen contributed to data interpretation. Chong Qiu and Junzhe Zhang performed mass spectrometry data acquisition. Ying Zhang, Peng Sun, Yin Kwan Wong, Qiaoli Shi, and Huan Tang assisted in other experiments and provided important suggestions. All authors contributed to manuscript revision and approved it for submission.

## CONFLICT OF INTEREST STATEMENT

The authors declare no conflict of interest.

## Supporting information


**Figure S1:** Determination of the antimalarial activity of APP and artesunate (ATS) on *P. falciparum* 3D7 strain.
**Figure S2:** In situ labeling of APP in live parasites in infected red blood cells.
**Figure S3:** Identification of the target proteins of artemisinin by APP through ABPP.
**Figure S4:** Protein–protein interaction (PPI) networks for all the 451 target proteins identified by APP‐based ABPP.
**Figure S5:** The rate of translation, peptide biosynthesis, and proteolysis processes throughout the entire IDC of *P. falciparum*.
**Figure S6:** Validations of the binding of ATS to target proteins including *PfTIM*, *PfGAPDH*, and *PfLDH*.
**Figure S7:** Determination of redox homeostasis‐related indicators in *P. falciparum* after ATS treatment and the validation of ATS targeting to *Pf1‐CysPxn* and *PfTrx‐Px1*.
**Figure S8:** Validations of the binding of ATS to glutathione GSH.
**Figure S9:** Partial least‐squares discriminant analysis (PLS‐DA) and metabolites differential analysis of *P. falciparum* treated with ATS versus control.
**Figure S10:** *Z*‐score analysis of the top 30 differential metabolites with significant differences ranked by *p* value.

**Table S1:** All target proteins identified by APP‐based ABPP in *P. falciparum* at three different stages of IDC.
**Table S2:** Metabolites of *P. falciparum* with significant differences after treatment with ATS versus DMSO.
**Table S3:** Metabolites and related proteins involved in enriched metabolic pathways.

## Data Availability

Research data are not shared. The data supporting this work this paper have been deposited in the OMIX, China National Center for Bioinformation/Beijing Institute of Genomics, Chinese Academy of Sciences (https://ngdc.cncb.ac.cn/omix/preview/2DszKpI6). The data and scripts used are saved in GitHub https://github.com/LabWang307/Antimalarial_Mechanism_ART. Supplementary materials (figures, tables, scripts, graphical abstract, slides, videos, Chinese translated version and update materials) may be found in the online DOI or iMeta Science http://www.imeta.science/.
