# Supplementary material for: Photoaffinity probe‐based antimalarial target identification of artemisinin in the intraerythrocytic developmental cycle of Plasmodium falciparum
Source: Imeta. 2024 Feb 19;3(2):e176. doi: 10.1002/imt2.176 (PMC11170969; doi:10.1002/imt2.176)
Supplement: Supplementary file 1 — Figure S1: Determination of the antimalarial activity of APP and artesunate (ATS) on P. falciparum 3D7 strain. Figure S2: In situ labeling of APP in live parasites in infected red blood cells. Figure S3: Identification of the target proteins of artemisinin by APP through ABPP. Figure S4: Protein–protein interaction (PPI) networks for all the 451 target proteins identified by APP‐based ABPP. Figure S5: The rate of translation, peptide biosynthesis, and proteolysis processes throughout the entire IDC of P. falciparum. Figure S6: Validations of the binding of ATS to target proteins including PfTIM, PfGAPDH, and PfLDH. Figure S7: Determination of redox homeostasis‐related indicators in P. falciparum after ATS treatment and the validation of ATS targeting to Pf1‐CysPxn and PfTrx‐Px1. Figure S8: Validations of the binding of ATS to glutathione GSH. Figure S9: Partial least‐squares discriminant analysis (PLS‐DA) and metabolites differential analysis of P. falciparum treated with ATS versus control. Figure S10: Z‐score analysis of the top 30 differential metabolites with significant differences ranked by p value. [file IMT2-3-e176-s001.docx]

**Supporting information to:**

**Photoaffinity probe-based antimalarial target identification of artemisinin in the intraerythrocytic developmental cycle of *Plasmodium falciparum***

**Running title:** Photoaffinity probe-based antimalarial target identification of artemisinin

Peng Gao^1,2#^, Jianyou Wang^3#^, Chong Qiu^1#^, Huimin Zhang^4#^, Chen Wang^1^, Ying Zhang^1^, Peng Sun^1^, Honglin Chen^3^, Yin Kwan Wong^1^, Jiayun Chen^1^, Junzhe Zhang^1^, Huan Tang^1^, Qiaoli Shi^1^, Yongping Zhu^1^, Shengnan Shen^1^, Guang Han^3^*, Chengchao Xu^1,2^*, Lingyun Dai^2^*, Jigang Wang^1,2,3,4,5^*

^1^State Key Laboratory for Quality Ensurance and Sustainable Use of Dao-di Herbs, Artemisinin Research Center, and Institute of Chinese Materia Medica, China Academy of Chinese Medical Sciences, Beijing 100700, China

^2^Department of Pulmonary and Critical Care Medicine, Shenzhen Institute of Respiratory Diseases, and Shenzhen Clinical Research Centre for Geriatrics, Shenzhen People's Hospital; First Affiliated Hospital of Southern University of Science and Technology; Second Clinical Medical College of Jinan University, Shenzhen 518020, China

^3^State key Laboratory of Antiviral Drugs, School of Pharmacy, Henan University, Kaifeng 475004, China

^4^Shandong Academy of Chinese Medicine, Jinan 250014, China

^5^Department of Oncology, the Affiliated Hospital of Southwest Medical University, Luzhou 646000, China

^#^These authors contributed equally: Peng Gao, Jianyou Wang, Chong Qiu, Huimin Zhang

*Correspondence: jgwang@icmm.ac.cn (Jigang Wang); lingyun.dai@outlook.com (Lingyun Dai); ccxu@icmm.ac.cn (Chengchao Xu); hang@henu.edu.cn (Guang Han)

1. **Supplementary Figures**

**Figure S1 Determination of the antimalarial activity of APP and artesunate (ATS) on *P. falciparum* 3D7 strain.** All data were based on at least three independent biological replicates and presented as the mean ± standard error of the mean (SEM).

**Figure S2** ***In situ* labeling of APP in live parasites in infected red blood cells.** (A) *In situ* labelling of APP in parasite with or without UV irradiation in a dose-dependent manner. (B) Labeling of APP target proteins in parasites when competed with excess ATS with or without UV irradiation. Coo, Coomassie brilliant blue.

**Figure S3 Identification of the target proteins of artemisinin by APP through ABPP.** (A) Scatter plots of target proteins of *P. falciparum* at different stages identified by APP with *in situ* ABPP experiments with or without UV irradiation. The results are displayed with the mean log_2_ ratio of the relative abundances between the APP (700 nmol/L) and DMSO groups (x-axis) against the -log_10_ of the *p* value (y-axis). Fold change (FC) > 1.3 and *p* value < 0.05 were used as the target selection criteria. (B) Heatmap representation of the abundance of target proteins identified by APP with and without UV irradiation at different stages of *P. falciparum*. Three biological replicates were conducted.

**Figure S4** **Protein-protein interaction (PPI) networks for all the 451 target proteins identified by APP-based ABPP.**

**Figure S5** **The rate of translation, peptide biosynthesis and proteolysis processes throughout the entire IDC of *P. falciparum*.** (A-B) Monitoring the translation and peptide biosynthesis of *P. falciparum* throughout the intraerythrocytic stage and the effect of ATS using AHA fluorescent labeling based on the ABPP method. (C-D) Determination of the proteolysis activity of *P. falciparum* at different stages with or without the treatment of ATS. The protease activity was measured using the Fluoro Protease Assay kit (G-Biosciences, C006028). OD, optical density. All data are averaged from three independent experiments and all values are presented as the mean ± standard error of the mean (SEM) (**p* < 0.05, ***p* < 0.01, ****p* < 0.01).

**Figure S6 Validations of the binding of ATS to target proteins including *PfTIM*, *PfGAPDH* and *PfLDH*.** (A-C) Validation of ATS to target proteins using the drug affinity responsive target stability (DARTS) strategy. (D-F) Determination of the inhibitory effect of ATS on the enzymatic activities of *PfTIM*, *PfGAPDH*, *PfLDH in vitro*, respectively. The results were calculated and expressed as the IC_50_ (Half maximal inhibitory concentration). All data were based on at least three independent biological replicates, and shown as the mean ± SEM. (G) Identification of the binding sites of ATS on the recombinant *PfGAPDH* protein and the corresponding docking simulation with - 5.0 (kcal/mol) binding energy.

**Figure S7 Determination of redox homeostasis-related indicators in *P. falciparum* after ATS treatment and the validation of ATS targeting to *Pf1-CysPxn* and *PfTrx-Px1*.** (A-B) ATS increases the levels of malondialdehyde (MDA) and reduces the peroxidase (POD) activity in a dose-dependent manner. (C) Fluorescence labeling of the recombinant *Pf*1-CysPxn and *Pf*Trx-Px1 proteins with APP under different conditions. ATS, artesunate; Flu, fluorescence; CBB, Coomassie brilliant blue.

**Figure S8** **Validations of the binding of ATS to glutathione GSH.** (A) Reaction scheme of GSH with ATS activated by heme. (B) Ultra-high performance liquid chromatography chromatograms of GSH and the adduct with ATS. (C) Mass spectrum of the adduct of ATS and GSH.

**Figure S9 Partial least squares discriminant analysis (PLS-DA) and metabolites differential analysis of *P. falciparum* treated with ATS versus control.** (A) PLS-DA plot of total parasite metabolites. (B) Volcano plot showing differential metabolites under the screening criteria with variable importance in projection (VIP) > 1, *p* values < 0.05, and FC > 1.2.

**Figure** **S10** **Z-score analysis of the top 30 differential metabolites with significant differences ranked by *p*-value.**
